# An Interesting Finding of Xanthogranulomatous Pyelonephritis Revealed Concomitant Incidental Squamous Cell Carcinoma (SCC) of the Renal Pelvis

**DOI:** 10.7759/cureus.63383

**Published:** 2024-06-28

**Authors:** Malik Samardali, Ibrahim Shanti, Jehad Samardaly, Leena Alhusari

**Affiliations:** 1 Internal Medicine, Marshall University, Joan C. Edwards School of Medicine, Huntington, USA; 2 Internal Medicine, Jordan University of Science and Technology, Ajloun, JOR

**Keywords:** renal keratinizing desquamating squamous metaplasia, renal pelvis tumor, chronic kidney disease (ckd), xanthogranulomatous pyelonephritis, renal squamous cell carcinoma

## Abstract

Squamous cell carcinoma, originating in the renal pelvis, is an infrequent form of kidney malignancy. The occurrence rate remains below 1% for all neoplasms in this specific area. Most of these carcinomas are moderately or poorly differentiated, and diagnosis typically occurs at an advanced stage. Xanthogranulomatous pyelonephritis is an uncommon form of severe chronic infection that affects the parenchyma of native kidneys. We present the case of a 34-year-old male with a history of end-stage renal disease secondary to recurrent pyelonephritis, which was incidentally diagnosed as renal squamous cell carcinoma (SCC).

## Introduction

Squamous cell carcinoma (SCC) originating in the renal pelvis is an infrequent form of malignancy, comprising less than 1% of all kidney tumors [1]. These carcinomas can be associated with chronic renal calculi, leading to squamous metaplasia and eventually squamous cell carcinoma [2,3]. Prolonged irritation, inflammation, and infection can trigger the development of squamous metaplasia within the renal collecting system, potentially advancing to dysplasia and carcinoma in the majority of affected individuals [4]. Typically, squamous cell carcinomas originating in the renal pelvis manifest as sizable, necrotic, and ulcerated lesions, displaying significant invasion into both the renal parenchyma and perinephric soft tissue [5]. These carcinomas are predominantly moderately or poorly differentiated and tend to be diagnosed at an advanced stage [4]. Even with efforts towards surgical removal and additional chemoradiotherapy, achieving a cure remains uncommon. The prognosis is bleak, with a less than 10% survival rate at the five-year mark. Xanthogranulomatous pyelonephritis is an uncommon form of severe chronic infection affecting the renal parenchyma in native kidneys that is diagnosed through ultrasonography or CT scan findings [6]. The simultaneous presence of keratinizing squamous cell carcinoma alongside xanthogranulomatous pyelonephritis is exceptionally uncommon, with only a few documented cases reported to date [7]. We present a case involving the coexistence of xanthogranulomatous pyelonephritis and primary squamous cell carcinoma of the renal pelvis, which was incidentally identified during pathological examination. Following an extensive review of existing literature, this case appears to represent the first documented instance of incidentally discovered squamous cell carcinoma without metastasis. Remarkably, it was managed solely through observation without the requirement for adjuvant chemotherapy.

## Case presentation

A 34-year-old male, born with congenital bladder exstrophy corrected at birth through internalisation of the external bladder, presented with complaints of severe right flank pain (rated 8 out of 10 in intensity) radiating into his back. These symptoms were accompanied by fever, nausea, and vomiting. He had a complex medical history, including end-stage renal disease due to recurrent urinary tract infections necessitating hemodialysis, chronic pyelonephritis requiring multiple nephrostomy tube insertions, and recurrent kidney stones. Blood tests and urine analyses were performed upon admission, and the findings are outlined in the tables provided below (Table 1, 2).

**Table 1:** Laboratory findings at initial presentation

Blood Tests	Results	Reference Range
White blood Cells count	15.34K/cmm	4.5-10 K/cmm
Hemoglobin	7.7 gm/dL	13-18 gm/dL
Platelet	310 k/cmm	150-450 k/cmm
Segmented neutrophil	84%	44.4-80%
Potassium	5.1 mEq/L	3.4-4.5 mEq/L
Sodium	158 mEq/L	135-145 mEq/L
Blood urea nitrogen	41 mg/dL	7-25 mg/dL
Creatinine	7.06 mg/dL	0.7-1.3 mg/dL

**Table 2:** Urinalysis

Urinalysis Dipstick	Result	Normal Findings
Clarity	Turbid	Clear or Cloudy
PH	8.0	5.00-8.00
Occult blood	Large	Negative
Glucose	500 mg/dL	Negative
Protein	200 mg/dL	Negative
Nitrate	Negative	Negative
Leukocyte esterase	Trace	Negative

**Figure 1:**
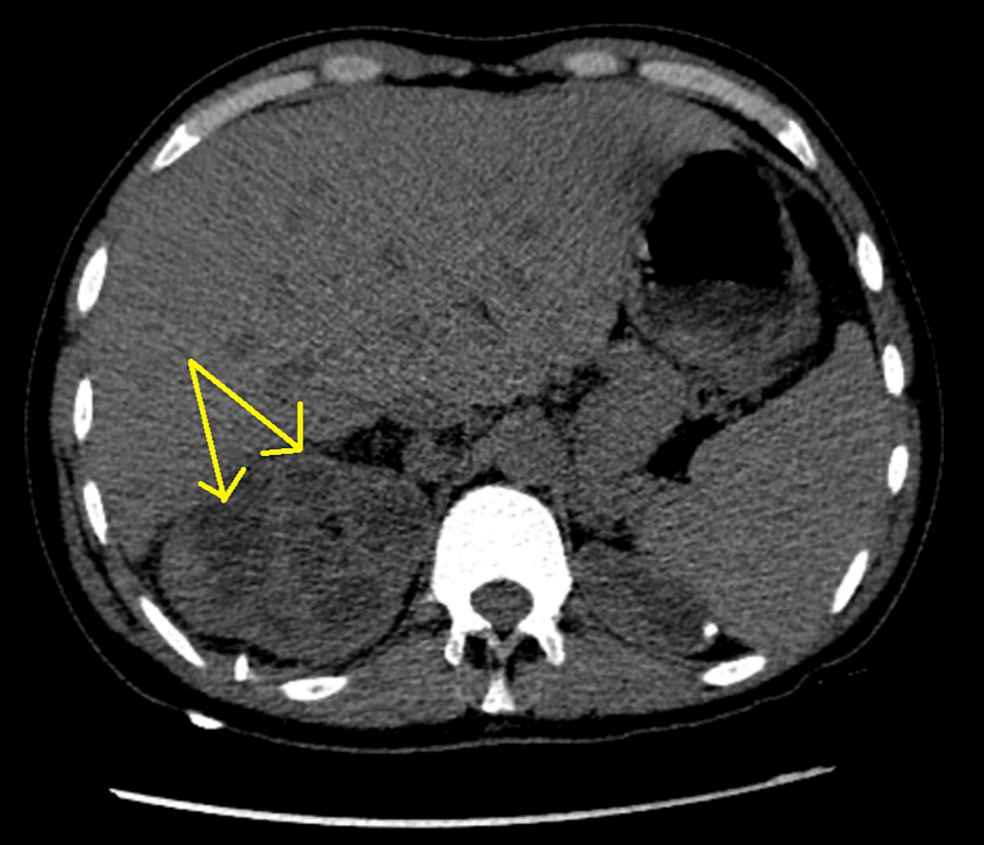
A non-contrast CT scan of the abdomen showed low-attenuation lesions scattered throughout the right kidney.

A CT scan of the abdomen without contrast revealed low-attenuation lesions throughout the right kidney, suggestive of xanthogranulomatous pyelonephritis (Figure 1).

**Figure 2:**
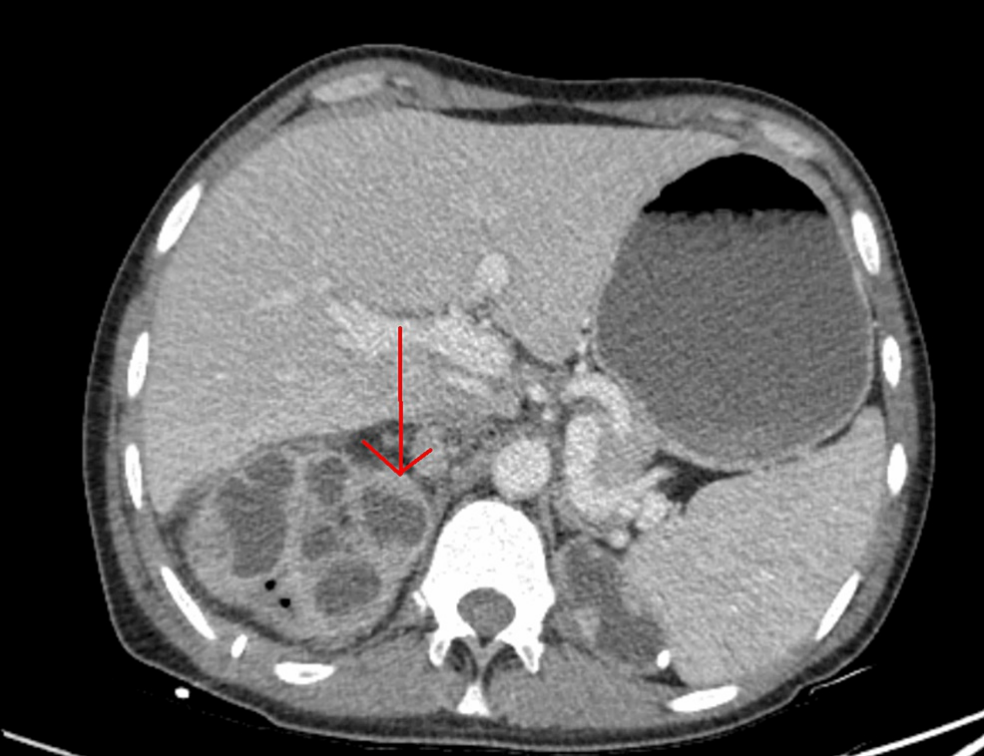
Contrast-enhanced abdominal CT scans revealed thickening of the right urothelium.

Abdominal CT scans with contrast showed persistent right urothelial thickening with moderate stranding around the kidney and ureter (Figure 2).

E. coli was detected in the urine culture, and the patient's condition improved following treatment with intravenous cefepime. Due to chronic pyelonephritis and the necessity to support future renal transplantation, a robotic-assisted laparoscopic right nephrectomy was performed following a request from nephrology to remove the kidney that had been subjected to multiple nephrostomy tubes.

**Figure 3:**
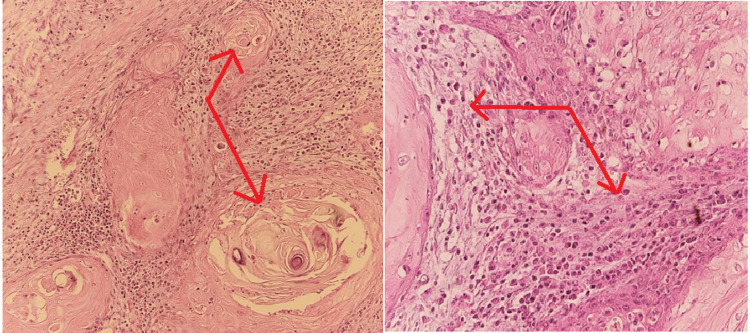
Microscopic examination. Histology shows a well-differentiated, invasive keratinizing squamous cell carcinoma of the renal pelvis. Representative images demonstrate muscularis invasion into the renal parenchyma with associated perineural invasion.

Microscopic examination of the right kidney specimens incidentally revealed a well-differentiated invasive keratinizing squamous cell carcinoma of the renal pelvis, invading through the muscularis into the renal parenchyma (Figure 3).

CT of the chest, abdomen, and pelvis with IV contrast revealed no evidence of metastasis. Following the National Comprehensive Cancer Network (NCCN) guidelines for treating squamous cell carcinoma of urinary tract origin, the hematology/oncology team opted for observation.

## Discussion

In the general population, squamous cell carcinoma accounts for 0.7% to 7% of all renal pelvic neoplasms [8]. Renal squamous cell carcinoma development may be associated with factors causing chronic inflammation, including calculi, infections, chemical exposure, vitamin deficiency, hormonal imbalance, and radiotherapy. It may also occur without any underlying factors [9]. Furthermore, prolonged irritation, inflammation, and infection resulting from these calculi can lead to squamous metaplasia of the epithelium in the renal pelvis, potentially advancing to squamous cell carcinoma (5). The histological cause and mechanism of renal parenchyma SCC are still unknown, despite the fact that squamous cell carcinoma of the renal pelvis was formerly thought to be urothelium metaplasia [10,11]. Squamous cell carcinoma of the kidney usually manifests at an advanced stage, typically at pT3 or higher. Surgery stands as the primary treatment modality for renal pelvic SCC. Adjuvant therapies offer limited benefits. Due to the advanced stage upon presentation, the prognosis tends to be unfavorable, given that surgical resection seldom achieves a curative outcome and adjuvant chemoradiotherapy is often ineffective. The prognosis is grim, with a five-year survival rate of less than 10% [12].

Xanthogranulomatous pyelonephritis (XGP), an uncommon variant of chronic pyelonephritis, typically arises due to chronic obstruction, resulting in hydronephrosis and subsequent renal parenchymal destruction [13]. Although often associated with lithiasis, XGP infrequently leads to keratinizing squamous metaplasia, closely resembling renal neoplasms and potentially resulting in misdiagnosis of malignancy [7]. Few cases of squamous cell carcinoma of the renal pelvis presenting as XGP have been documented in the literature [7,14,15]. Studies have reported cases where squamous cell carcinoma of the kidney parenchyma mimicked XGP on radiological examination [16]. Histopathological evaluations have shown cases of XGP coexisting with squamous cell carcinoma and osteogenic sarcoma of the kidney [17]. XGP is characterized by the presence of lipid-laden, foamy macrophages and inflammatory cells in the renal parenchyma [18]. XGP can present as a focal disease process, mimicking renal cell carcinoma in some cases [19]. The clinical, radiographic, and angiographic presentations of XGP can often be mistaken for renal cell carcinoma, as it has been reported that XGP is frequently misdiagnosed as renal carcinoma due to its presentation and radiographic appearance [20].

In our case, an abdominal CT scan without contrast showed low-attenuation lesions throughout the right kidney, which was suggestive of xanthogranulomatous pyelonephritis (XGP). The radiological presentation of squamous cell carcinoma of the renal pelvis can vary and may include features such as diffuse enlargement of a nonfunctional kidney, the presence of renal calculi, perirenal infiltration, and areas of low density or echogenicity within the renal parenchyma. Differentiating primary renal squamous cell carcinoma from conditions such as xanthogranulomatous pyelonephritis or other malignant kidney neoplasms can be challenging.

## Conclusions

Primary squamous cell carcinoma of the renal pelvis is not commonly reported in the literature. XGP infrequently leads to keratinizing squamous metaplasia, closely resembling renal neoplasms, and potentially results in the misdiagnosis of malignancy. Our case presents a concomitant occurrence of squamous cell carcinoma of the renal pelvis and XGP, which has rarely been reported in the literature.
